# Parents’ perceptions of barriers & facilitators for adhering to advice from community health nurses

**DOI:** 10.1093/eurpub/ckac131.437

**Published:** 2022-10-25

**Authors:** RR Carlsson, LAS Brautsch, L Kierkegaard, CT Bonnesen

**Affiliations:** National Institute of Public Health, Copenhagen, Denmark

## Abstract

**Background:**

Childhood obesity is a global health problem and infancy is an important window of opportunity for promoting healthy weight development. In Denmark, community health nurses cover most families with a new-born child leaving a huge potential for promoting healthy weight development. This study examines parents’ perceptions of barriers and facilitators for adhering to advice from the health nurse regarding healthy weight development of their child.

**Methods:**

Sixteen interviews with parents (children aged 0- 2 years) living in Denmark were conducted. Parents were strategically sampled representing variations across child age, family socioeconomic position, ethnicity, and geography. All interviews were based on a semi-structured interview guide, recorded, and transcribed verbatim and analysed using a thematic analysis.

**Findings:**

Parents generally experience visits from their health nurse positively. Health nurses are emphasized as a confident and trustful relation and the key informant about children's’ health. Variations in how parents adhere to advice favouring healthy weight development of their child were found. Generally, parents adopted a positive attitude towards the health nurse, also when she addressed behaviour or practice of the parents, but parents expressed the importance of doing so in a non-stigmatising or finger pointing manner. The degree to which parents follow the advice from their health nurse depend on cultural background, advice from family and friends, and use of online information.

**Conclusions:**

Parents generally have a confident and trustful relation to the health nurse, and she constitutes the key informant on child health. This leaves a potential for the structure of Danish health nurses for future interventions promoting healthy weight development. Adaptation of the future intervention program to the needs of parents will increase the chances of developing a relevant, successful, and sustainable intervention.

**Key messages:**

• Health nurses are emphasized as a trustful relation and the key informant about children’s’ health, but parents expressed the importance of addressing behaviours in a non-stigmatising manner.

• Adapting information gained from parents in development of the trial will increase the chances of a relevant, successful, and sustainable intervention promoting healthy child weight development.

